# Necrotizing Fasciitis: A Life-threatening Complication of Intraoperative Electromyography

**DOI:** 10.7759/cureus.468

**Published:** 2016-01-25

**Authors:** Alireza Shoakazemi, Marc Moisi, R. Shane Tubbs, Mary Wingerson, Olaide Ajayi, Michael E Zwillman, Jourdan Gottlieb, David Hanscom

**Affiliations:** 1 Neurosurgery, Swedish Neuroscience Institute; 2 Neurosurgery, Seattle Science Foundation; 3 Anesthesiology and Critical Care, Houston Methodist Hospital; 4 Plastic Surgery, Swedish Medical Center

**Keywords:** intraoperative neurophysiological monitoring, soft tissue infection, needle electrode infection, necrotizing fasciitis, spine surgery, emg

## Abstract

Intraoperative neurophysiological monitoring is a commonly used practice during spine surgery. Complications of electromyography (EMG) needle electrode placement are very uncommon. We present a patient who was infected with necrotizing fasciitis in her left thigh due to an EMG needle electrode during a two-stage complex spine procedure. This case demonstrates that providers should continue to meticulously adhere to protocol when inserting and removing EMG needles, but also be cognizant during postoperative observation of the possibility of infection caused by EMG needles.

## Introduction

The common use of intraoperative neurophysiological monitoring, such as electromyography (EMG) for spine surgical procedures, requires the placement of needle electrodes. The needles are placed by the neurophysiology technologist or by the surgeon under clean but not sterile techniques. Complications of EMG needle placement include bleeding, nerve injury, pneumothorax, or electrical injury [1]. Soft tissue infections related to needle placement are exceptionally rare.

We present a rare case of a deep soft tissue infection after insertion of an intraoperative electromyographic monitoring needle. The outcomes of this case, as well as a review of the literature, are discussed.

## Case presentation

A 57-year-old female presented with left-sided leg pain and swelling after a two-stage complex spine operation. The patient was suffering from chronic back pain, right leg pain, and neurogenic claudication. On evaluation, she was found to have degenerative scoliosis as well as central and foraminal stenosis. Initially, the patient underwent an L3-S1 anterior lumbar interbody fusion, followed by a second stage L3 to the sacrum posterior spinal fusion and extraforaminal nerve root decompression two weeks later. Informed patient consent had been obtained prior to her treatment.

In the second stage of the procedure, we used a commercially available electromyographic (EMG) intraoperative monitoring system. Using electromyography monitoring is routine practice for lumbar spinal fusions in our department. Disposable EMG needles were inserted after the area was cleaned with alcohol wipes. These needles are 22 mm in length and longer than standard acupuncture needles and other EMG monitoring systems (typically 13-14 mm long). This needle length was used as part of one our vendor's monitoring packages and unrelated to the patient's body habitus. The needles were secured in place using tape. During the procedure, there was no need to reposition any of the needles due to dislodgment or other technical issues. We removed the needles at the end of the procedure while the patient was still in the prone position. The patient had an uneventful immediate postoperative period except for mild hypotension and was discharged home three days after the procedure.

At a routine follow-up clinic visit at nine days after the second stage procedure, the patient reported a few episodes of fever up to 101^o^F. She also complained of bilateral leg swelling with pain and heaviness on both sides. She reported that elevation of both legs did not help her symptoms. Due to ongoing leg pain and swelling, ultrasonography (US) of both legs was performed, which excluded deep venous thrombosis. In the three week period following her second stage surgery, her leg pain and swelling deteriorated to the extent that she was unable to move her left leg. Physical examination of her legs at this time showed evidence of bilateral non-pitting edema, which was more pronounced in the left leg. There was no evidence of erythema or purulent discharge at this visit. A repeat lower extremity US did not show any evidence of deep venous thrombosis.

**Figure 1:**
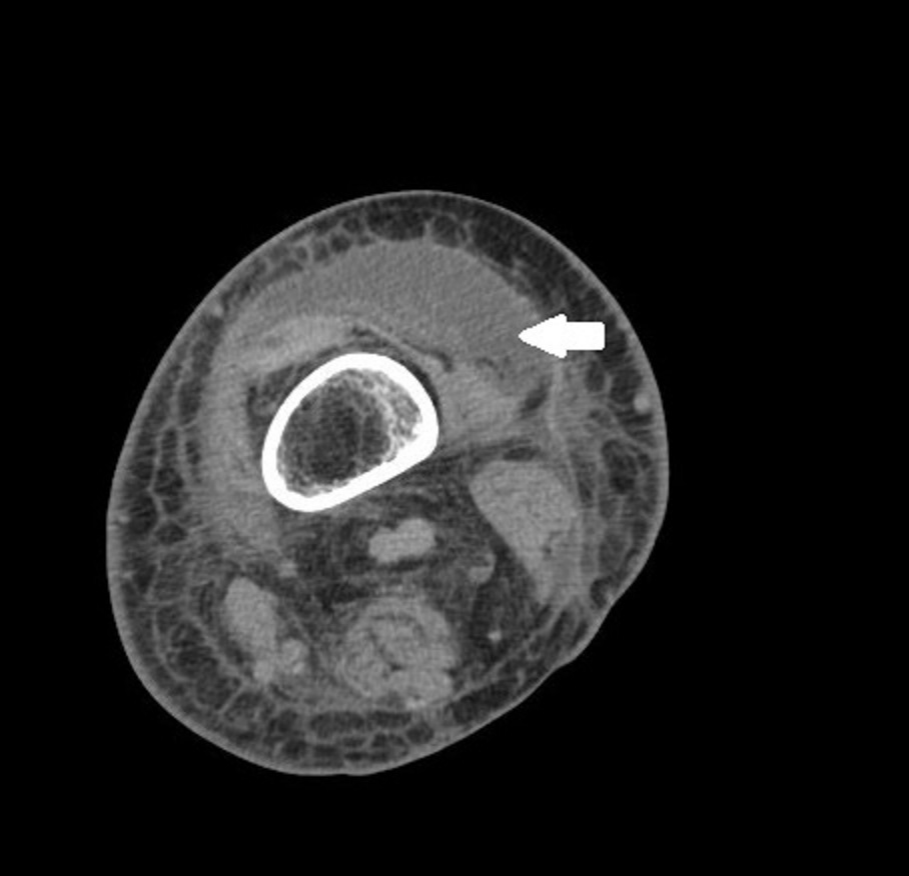
Axial CT Scan of Left Lower Extremity Arrowhead demonstrates discrete intramuscular fluid collection with the largest focus centered in the vastus lateralis.

**Figure 2:**
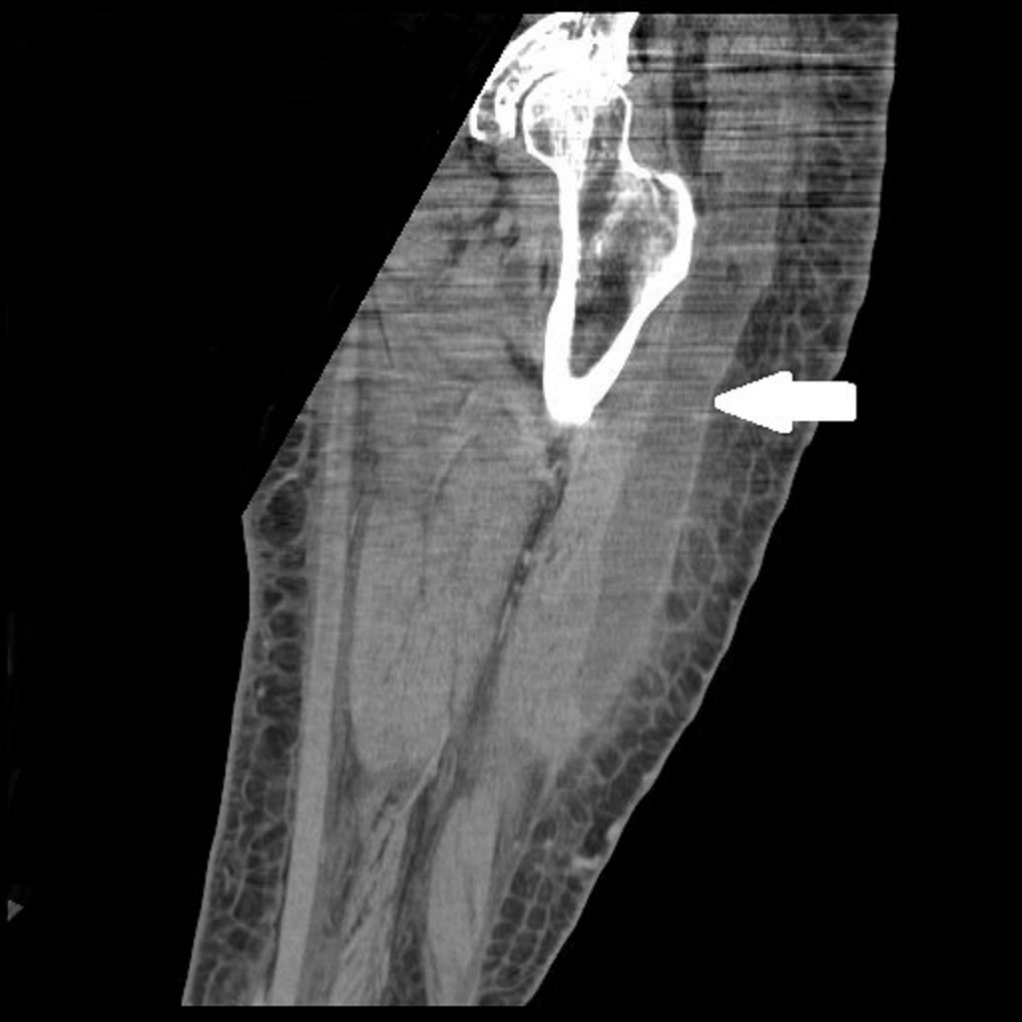
Coronal CT Scan of Left Lower Extremity Arrowhead demonstrates discrete intramuscular fluid collection with the largest focus centered in the vastus lateralis.

Her left leg swelling increased and she required readmission almost 29 days after her second stage surgery. At this time, she also developed significant hyponatremia (124 mEq/L). On this presentation, she was normothermic with bright erythema from the knee to groin with extension to the pelvis. She showed blotchy red areas in both feet. The erythematous area of her left leg was tender on palpation. She denied use of any antibiotics prior to this admission. C-reactive protein was elevated to 33 mg/L on this presentation. US and CT of both legs showed areas of un-opacified muscle, inflammation, and possible fluid collection in the left thigh. The CT findings are demonstrated in Figures 1-2.

Her clinical presentation, laboratory data, and CT imaging raised a concern about a deep soft tissue infection. She was taken to the operating room urgently for aspiration of left thigh purulent fluid and excision and debridement of left thigh necrotic fascia and muscle. During this procedure, 900 cc of purulent fluid were drained and the vastus lateralis muscle was found to be necrotic. A wound vac (negative pressure wound therapy) was applied at the end of the procedure. Broad-spectrum antibiotics, which included vancomycin, clindamycin, and Zosyn (piperacillin and tazobactam), were started immediately. Her antibiotic therapy was changed to Unasyn (ampicillin sodium/sulbactam sodium) after tissue cultures grew *Bacteroides fragilis*. Following the fasciotomy, she developed a psoas abscess followed by a lumbar spine wound dehiscence, both of which required further debridements. After further debridement of the left hip, flank, pelvis, retroperitoneum, and left leg, the wound was closed as a separate procedure.

## Discussion

Necrotizing fasciitis (NF), a rare and life-threatening infection of deep soft tissues and fascia, is rarely reported as a consequence of needle electromyography [2]. Some other similar procedures, e.g. acupuncture, have been previously reported as a cause of a deep soft tissue infection, such as necrotizing fasciitis [2-3]. Infectious organisms due to acupuncture needles have included *Escherichia coli* and *Mycobacterium tuberculosis* [4]. NF classically presents with subcutaneous edema, ecchymosis, purpura, hemorrhagic bullae that can rapidly evolve into vascular occlusion with resultant tissue ischemia, necrosis, and gangrene. However, in this unusual case, the atypical initial presentation required a higher index of suspicion. In many cases, it may be difficult to make a definitive diagnosis of NF based only on physical examination findings [3]. Factors, such as peripheral WBC, serum sodium, and blood urea nitrogen, can assist in the diagnosis of deep soft tissue infections [3]. In our case, clinical suspicion and laboratory findings, such as hyponatremia, were helpful to make the diagnosis. She did not have any other risk factors, including obesity (BMI 16.4), diabetes, or immunosuppression.

The American Association of Neuromuscular and Electrodiagnostic Medicine (AANEM) position statement insists on practitioners' knowledge about potential complications of electrodiagnostic medicine to minimize transmission of pathogens during this procedure. Although this guideline mainly focuses on blood-borne pathogen transmission, adherence to strict criteria in skin preparation and insertion of needles can reduce the occurrence of similar complications. Use of universal precautions, such as gloves and mask, are strongly advised in all cases. Needle EMG is not listed in the American Heart Association guidelines as a procedure which mandates prophylactic antibiotic treatment to prevent endocarditis in the background of valvular heart disease [5]. However, in most intraoperative monitoring cases, prophylactic antibiotics are administered as a part of the main operation protocol. Alcohol has been recognized as an acceptable method of cleaning the skin prior to insertion of EMG needles, although no data is available that skin preparation in this particular procedure reduces infection [5]. Use of needle electrodes in the setting of an edematous tissue should be practiced with caution since chronic weeping of serous fluid from the puncture site can act as a potential bacterial media and increase the risk of cellulitis [5].

Previously, EMG needle-related soft tissue infections were mostly due to reusable needles, which are rarely used nowadays. Nolan, et al. reported an outbreak of infections due to *Mycobacterium fortuitum* associated with EMG needles in six patients [6]. In this report, needles were reused for other patients. Although NF secondary to acupuncture with causative organisms, such as *Pseudomonas aeruginosa, Staphylococcus aureus, Enterococcus faecalis*, gram negative rods, and*Vibrio vulnificus,* have been reported in the literature, in general, infectious complications of acupuncture are deemed extremely rare [2]. In the case reported herein, the use of disposable needles and 70% alcohol wipes was according to our hospital’s protocol. It is unclear whether the length of the EMG needles may have been a contributing factor.

We recommend adherence to a strict protocol and universal precautions during insertion and removal of EMG needles to minimize the occurrence of similar complications. Considering the rarity of this complication, it is unclear whether commercially available methods of EMG monitoring and size of the EMG monitoring needles can have a deleterious effect on inoculation of organisms to deeper tissue layers during insertion. In similar cases, a high level of suspicion in similar cases and a thorough investigation by a specialist in this field can detect soft tissue infection at an early stage and prevent the devastating consequences of NF.

## Conclusions

Although this case is a rare instance of a severe multifocal deep, soft tissue infection most possibly related to an electromyography needle, we are not sure if this mandates a change in practice concerning insertion or preparation of the area of needle insertion. Greater attention to the cleanliness of this procedure and meticulous skin preparation with perhaps alcohol cleaning of the actual EMG needle can prevent any potential similar complications in the future. We advise a high level of suspicion in similar cases to detect EMG needle-related soft tissue infection at an early stage.
